# Supplementary figures and images for: Diisocyanates influence models of atopic dermatitis through direct activation of TRPA1
Source: PLoS One. 2023 Mar 6;18(3):e0282569. doi: 10.1371/journal.pone.0282569 (PMC9987805; doi:10.1371/journal.pone.0282569)

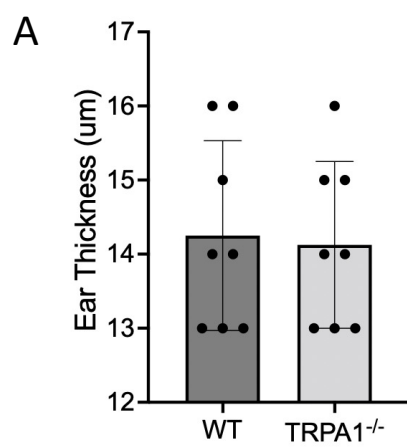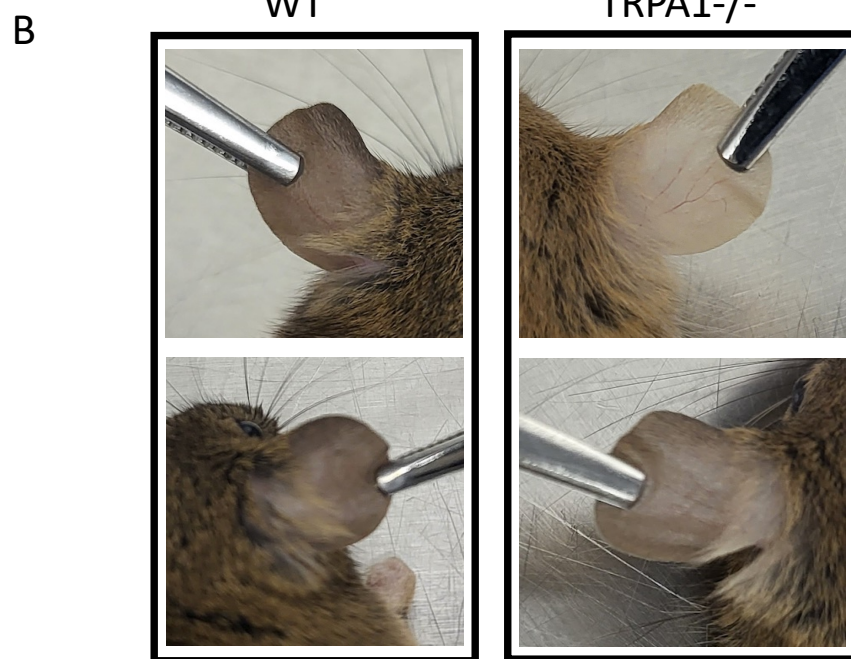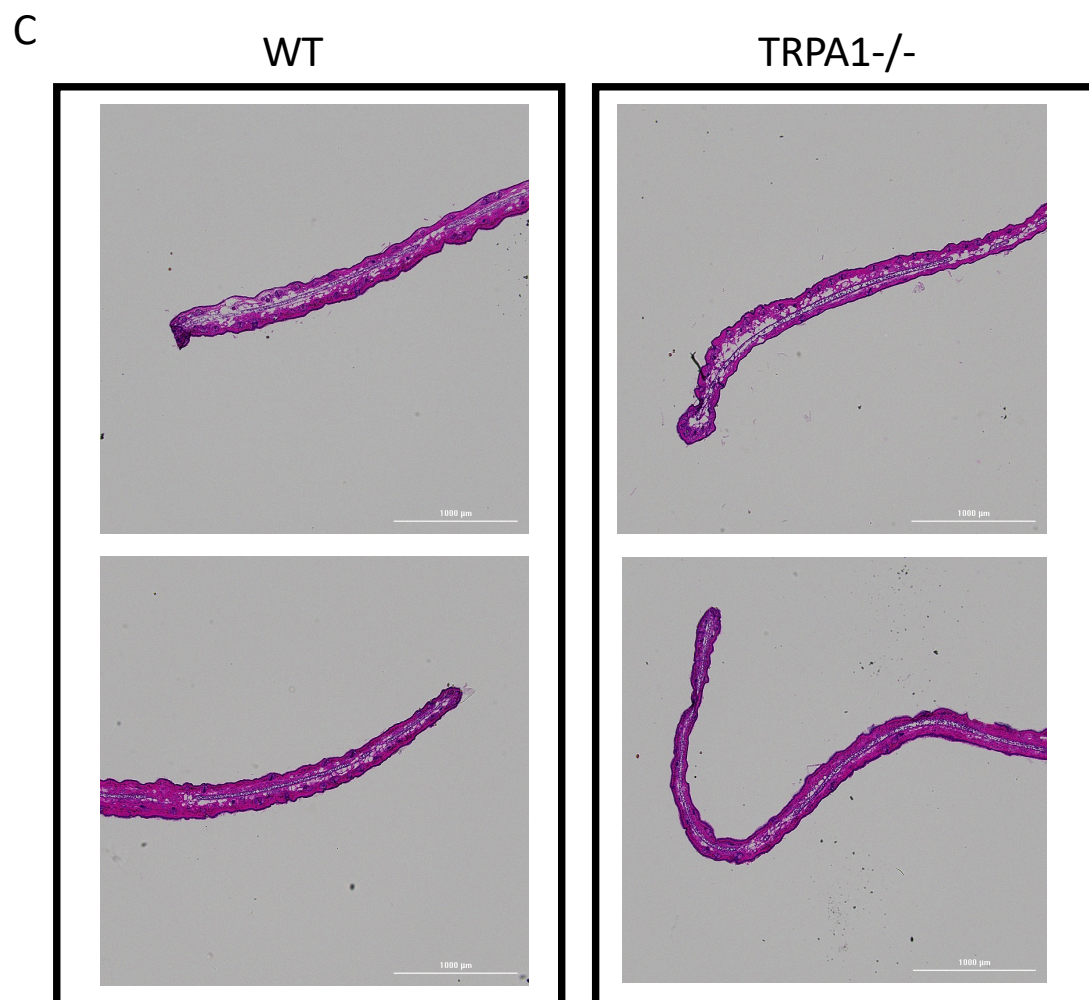

Supplement: S1 Fig — (A) Left ear thickness from 8 mice aged 8 weeks for either WT or TRPA1-/- mice. (B) Representative images from two mice per group showing gross ear anatomy. (C) Representative H&E slides from two mice per group demonstrating baseline histology. (PDF) [file pone.0282569.s002.pdf]
