# Supplementary material for: Diisocyanates influence models of atopic dermatitis through direct activation of TRPA1
Source: PLoS One. 2023 Mar 6;18(3):e0282569. doi: 10.1371/journal.pone.0282569 (PMC9987805; doi:10.1371/journal.pone.0282569)
Supplement: Attachment — Submitted filename: Reviewer replies v2.docx [file pone.0282569.s003.docx]

Reviewer replies

Reviewer #2: My overall impression is that the authors did not pay much attention to this manuscript. Fig. 2 and 3 are mistakenly cited in the text and data in Fig. 4 and 5 are poorly organized, which all made the manuscript difficult to read and follow.

To improve the scientific quality of this paper, I suggest the authors to provide more results in Fig. 1 as follows, and reorganize the data and description of Fig. 2-5 to make it readable and meaningful.

We made the error of mislabeling Fig 2 and 3 (they were switched in the upload). The call outs were correct, but we apologize and understand why this flip was confusing. By placing them in proper order we now have the intended flow of

1 – Establishing TRPA1 mice do not have as severe of AD-like dermatitis after TDI exposure

2 – Blocking TRPA1 improves outcomes in the MC903 model of AD

3 – TRPA1 deletion alters metabolism

4 – TRPA1 blockade alters metabolism

5 – The altered metabolism established in 3 and 4 collate into tyrosine metabolism, which can be further targeted by cell culture model.

Fig. 1B-D: Results from WT and TRPA1-/- mice before TDI treatment should be provided. If there is no direct comparison of mouse phenotypes before and after TDI treatment (essentially on the same ears of mice), how could the authors conclude that TDI can induce atopic dermatitis and TRPA1 has a contribution? Even if the TDI-induced model has been established previously, these works should not be neglected as they are important and very easy to do.

We now present data from WT and TRPA1-/- mice without TDI exposure. As has been established in the prior use of these animals – there are no obvious baseline differences. We understand the request for baseline difference, but seeing none we continue to conclude that this established model is correct and that the TDI exposure’s failure to induce a similar degree of inflammation in the knockout mice supports our conclusion that TDI-induced dermatitis is partially dependent on TRPA1.

Fig. 1E-F: The authors claimed direct activation of TRPA1 by TDI, however the data from Schwann cells are not sufficient to support this conclusion. Patch clamp (ideally) or calcium imaging on TRPA1-overexpreesing and control cells should be performed and a dose-response curve of TDI is essential.

We now present three different doses of TDI to demonstrate the dose response curve requested and thank the reviewer for this recommendation. However, we continue to present data that the calcium influx is dependent on TRPA1. Overexpressing TRPA1 would not impact these conclusions. If overexpression of TRPA1 increased flux, indeed that would support the notion that it was involved – but failure to do so would not comment on TRPA1s role since the rate limiting step of calcium influx may not be the level of receptor expression. Our findings are clear – adding TDI in the presence of a specific TRPA1-blocker negates calcium influx. Given that we now also show that this occurs in a dose dependent manner, we do not feel that the patch clamp or overexpression would change the conclusions presented (save a claim that Ca influx would not carry an obvious change in conductivity).

Fig. 1F: What does Pre-Tx mean? It does not seem to be a typo of HC but something else.

Tx is an established abbreviation for “treatment” for physicians, but this was left in error. We apologize and have fixed the figure, so it is Pre-HC to match the remainder of the figure.

Editor replies:

Abstract: The abstract is poorly presented. Too long background information (line 13-line 24). The authors should rewrite it with concise background, methodology and results, and conclusions/implications

The background portion of the abstract has been amended.

Line 69, “10mcL per ear was applied to”.  What unit? How to apply?

We are not sure what is being asked by “what unit” since mcL is a well established unit of volume, but we have spelled out microliter and added “topically” to the description of how this was applied “to the ear”.

Line 81, “t” not “T”, also line 165

This has been changed

Line 82, abbr. “IACUC” should give full name. Check whole manuscript for similar the issue.

This has been added

Line 87, subscript “2” for CO2

This has been changed

Line 95, leave one space between number and unit

This has been changed

Line 102, Should be “Calcium influx …”

This has been changed although technically calcium outflux would also be visible, thus “flux” seemed the most appropriate term.

Line 112, change the subheading “bacteriology” and give a proper subheading

We changed this to “bacterial culture”

Line 119, abbr. “MALDI”?

This has been added

Line 130, Abbr.: NMDS

This has been added

Results

There are some errors for citing figure numbers (see reviewer comments as well). The result description should be clear and detailed. The paragraph for each result section should be treated in one figure. The figure 5 seems to be for the text under two subheadings, and the "signalling data" section was listed as half figure in figure 5e-g.

We made the error of mislabeling Fig 2 and 3 (they were switched in the upload). The call outs are correct, but we apologize and understand why this flip was confusing. We have moved the section on figure 5 to one paragraph. We have left Fig 3 and 4 combined because they are highly related and are only separate because combining them would render the text too small to read.
